# Trends in Annual Sales and Pack Price of Cigarettes in the US, 2015-2021

**DOI:** 10.1001/jamanetworkopen.2022.15407

**Published:** 2022-06-03

**Authors:** Fatma Romeh M. Ali, Elizabeth L. Seaman, Barbara Schillo, Donna Vallone

**Affiliations:** 1 CDC Foundation, Atlanta, Georgia; 2 Truth Initiative, Washington, DC

## Abstract

This cross-sectional study assesses trends in total cigarette retail sales, menthol cigarette sales, and cigarette prices in the US from 2015 to 2021.

## Introduction

A 2020 Federal Trade Commission (FTC) report^1^ revealed that the number of cigarettes sold to wholesalers and retailers had increased for the first time in 2 decades, prompting concern about a potential reversal to the 30-year decline in cigarette use in the US^2^ However, these data did not reflect actual sales to consumers. National surveillance studies have indicated a decline in cigarette use among youth^3^ and adults.^4^ To examine cigarette market changes in relation to actual consumer purchases, this cross-sectional study assessed trends in total cigarette retail sales, menthol cigarette sales, and cigarette prices in the US from 2015 to 2021.

## Methods

Cigarette sales data were licensed from IRi, which records brick-and-mortar retail scanner sales but not online sales (eMethods in the Supplement). Sales of 20-cigarette packs^5^ were aggregated annually from February 1, 2015, to December 26, 2021, for the continental United States and Washington, DC.

Trends in total pack sales, menthol share from total pack sales, total dollar sales, and pack price were assessed using Joinpoint Regression Program, version 4.9.0.0 (National Cancer Institute). Average annual percentage change (AAPC) and 95% CIs were calculated. Statistical significance was defined as 2-tailed *P* < .05. This cross-sectional study followed the STROBE reporting guideline. This study was approved by a central institutional review board, Advarra, which determined that institutional review board oversight was not required because it did not involve human participants research.

## Results

**Figure 1.**
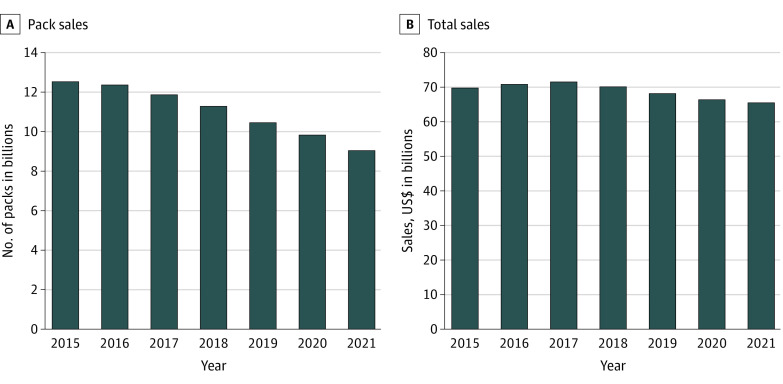
Cigarette Sales in the Continental United States, 2015-2021 Represents sales from convenience stores, gas stations, grocery stores, drug stores, mass merchandiser outlets, retail chain stores, club stores, dollar stores, and military sales.

**Figure 2.**
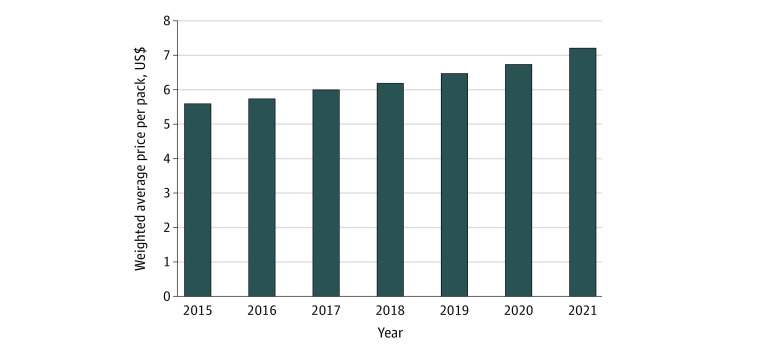
Weighted Average Price per Cigarette Pack in the Continental United States, 2015-2021

From February 1, 2015, to December 26, 2021, the annual number of cigarette packs sold decreased by 27.2%, from 12.5 billion packs to 9.1 billion packs (AAPC, −5.3%; 95% CI, −6.6% to −3.9%) (Figure 1A). During this period, dollar sales decreased overall by 6.0%, from $69.8 billion to $65.6 billion (AAPC, −1.2%; 95% CI, −2.0% to −0.3%) (Figure 1B), while prices increased by 29.5%, from $5.57 to $7.22 per pack (AAPC, 4.3%; 95% CI, 4.1% to 4.6%) (Figure 2). Menthol cigarettes accounted for 33.0% of total sales in 2021; there was no significant change in this percentage from 2015 to 2021.

From January 26, 2020, to December 26, 2021, annual cigarette pack sales decreased by 7.6% (9.8 billion to 9.1 billion packs); dollar sales decreased by 1.4% ($66.5 billion to $65.6 billion), while prices increased by 6.8% ($6.76 to $7.22 per pack). Menthol share remained stable at 33.0%.

## Discussion

From February 1, 2015, to December 26, 2021, total annual cigarette retail sales decreased significantly in both dollars and units. Menthol share was stable at 33%, suggesting that the decline in sales was consistent across menthol and nonmenthol cigarettes. The difference in sales trends from the FTC data^1^ could be explained by differences between the 2 data sources. The FTC data reflect cigarettes sold by major manufacturers to wholesalers and distributors within the US and to armed forces personnel stationed outside the US,^1^ whereas retail sales data reflect actual consumer purchases at the point of sale within the US. Furthermore, unlike retail sales data, the FTC data may include online sales and sales in Alaska and Hawaii because distinctions based on location or type of retailers are not made. However, online cigarette sales were restricted after passage of the Prevent All Cigarette Trafficking Act of 2009.^6^ The decline in retail cigarette sales is consistent with the continuing decline in cigarette use among youth^3^ and adults^4^ in the US.

Both the FTC and retail sales data are likely associated, in part, with recent challenges associated with the COVID-19 pandemic and its effects on the global economy, consumer purchase patterns, and the supply chain. Retail sales data suggest that prices increased during the study period. However, it is unclear whether retail sales are lower because prices are higher, which might reflect increases in production costs, or whether retailers increased the prices to offset the loss of revenue from low demand associated with the pandemic.

Findings from this analysis are limited in that retail sales do not reflect actual consumption. In addition, this analysis is descriptive, and causal relationships cannot be inferred. Nevertheless, continued surveillance of tobacco sales provides timely insights into emerging trends in consumer product preferences and can inform policy planning and practice.

## Notes

Supplement.
eMethods.

eReferences
