# Supplementary material for: Trends in Annual Sales and Pack Price of Cigarettes in the US, 2015-2021
Source: JAMA Netw Open. 2022 Jun 3;5(6):e2215407. doi: 10.1001/jamanetworkopen.2022.15407 (PMC9166220; doi:10.1001/jamanetworkopen.2022.15407)
Supplement: Supplement. — eMethods. eReferences [file jamanetwopen-e2215407-s001.pdf]

Supplemental Online Content

Ali FRM, Seaman EL, Schillo B, Vallone D. Trends in annual sales and pack price of cigarettes in the US, 2015-2021. *JAMA Netw Open*. 2022;5(6):e2215407.
doi:10.1001/jamanetworkopen.2022.15407

eMethods.

eReferences

This supplemental material has been provided by the authors to give readers additional information about their work.

© 2022 Ali FRM et al. *JAMA Network Open.*

eMethods

Cigarette retail sales data were licensed from IRI (Chicago, IL), Inc., which reflect data from a sample of convenience stores, gas stations, grocery stores, drug stores, mass merchandiser outlets, retail chain stores, club stores, dollar stores, and military sales, for the 48 continental states (excluding Alaska and Hawaii). Sales from online retailers are not available. The data included weekly sales (in units and dollars) for each universal product code and product features such as flavor and total count of cigarettes per pack.

This study aims to assess trends in cigarette retail sales during the period presented in the recent FTC report (2019-2020). However, we expanded the study period to include years from 2015 to 2021 to ensure enough sample size to conduct a trend analysis using Joinpoint Regression Program (version 4.9.0.0, National Cancer Institute).

Following previous studies, cigarette unit sales were standardized so that one unit equals one pack (20 cigarettes).[1,2] Sales were summed annually during 2015-2021. Weighted average price per pack (referred to as "price" in the text) was calculated as total dollar sales divided by total pack sales. Menthol unit share was calculated as the percentage of menthol cigarette pack sales from total pack sales. Joinpoint regression was used to quantify the direction and magnitude of changes in trends of total sales, menthol share, and price per cigarette pack.

eReferences

1. Marynak KL, Gammon DG, King BA, et al. National and State Trends in Sales of Cigarettes and E-Cigarettes, U.S., 2011-2015. *Am J Prev Med*. 2017;53(1):96-101. doi:10.1016/j.amepre.2017.01.016

© 2022 Ali FRM et al. *JAMA Network Open.*

2. Kuiper NM, Gammon D, Loomis B, et al. Trends in Sales of Flavored and Menthol Tobacco Products in the United States During 2011-2015. *Nicotine Tob Res*. 2018;20(6):698-706. doi:10.1093/ntr/ntx123

© 2022 Ali FRM et al. *JAMA Network Open.*
